# Congenital nephrotic syndrome associated with 22q11.2 duplication syndrome in a Chinese family and functional analysis of the intronic NPHS1 c. 3286 + 5G > A mutation

**DOI:** 10.1186/s13052-019-0690-2

**Published:** 2019-08-23

**Authors:** Liangliang Li, Zhi Yi, Hongmin Xi, Lili Ma, Hui Shao, Wenwen Wang, Hong Pan, Miaomiao Li, Hong Jiang

**Affiliations:** 1 Neonatal Department, The Affiliated Hospital of Qingdao University, NO.16 Jiangsu Road, Shinan District, Qingdao, 266003 Shandong China; 2 Neurological and Endocrine Department of Pediatric Center, The Affiliated Hospital of Qingdao University, Qingdao, China; 3 Intensive Care Unit, The Affiliated Hospital of Qingdao University, Qingdao, China; 4 Department of Central Laboratory, Peking University First Hospital, Beijing, China; 5 Medical Genetic Department, The Affiliated Hospital of Qingdao University, Qingdao, China

**Keywords:** Congenital nephrotic syndrome, 22q11.2 duplication syndrome, NPHS1, Minigene assay

## Abstract

**Background:**

Congenital nephrotic syndrome (CNS), which is defined as heavy proteinuria, hypoalbuminemia, hyperlipidemia and edema, is most caused by monogenic defects in structural proteins of the glomerular filtration barrier in the kidneys. 22q11.2 duplication syndrome was a chromosomal disease with variable clinical featuresranging from normal to mental retardation and with congenital defects. Co-occurrence of two genetic disorders in a single patient is rare.

**Case presentation:**

The proband was born at 36 weeks of gestational age spontaneously and weighed 2350 g at birth. Six days after birth, the proband was admitted to our hospital due to fever of 38.5 °C lasting for 6 h. Physical examination at admission time showed dysmorphic features of hypertelorism, palpebral edema, broad nose bridge, upturned nose, dysmorphic auricle, long philtrum, and a thin upper lip. Additionally, we found left wrist drop and bilateral strephexopodia, bilateral knee joint flexion contracture in this patient. A series of indicators were detected and showed abnormalities. Albumin was used to remit the hypoproteinemia and edema. However, the parents refused to accept further therapy and the boy died at age 3 months due to cachexy. To confirm the pathogenesis, genetic analysis were performed and revealed two mutations of NPHS1 gene: Exon18: c.2386G > C; p. (Gly796Arg) inherited from mother, and intron24: c.3286 + 5G > A; p.? inherited from father. And he also had a 22q11.2 duplication which was inherited from his mild affected mother. The pathogenesis of the intronic mutation has been further identified that it can defect alternative splicing of NPHS1.

**Conclusions:**

We present a patient who was caught in congenital nephrotic syndrome and 22q11.2 duplication syndrome simultaneously, emphasizing the importance of new sequencing technology on diagnosis of different genetic disorders.

## Background

Nephrotic syndrome (NS) is a rare disorder characterized clinically with the presence of edema and biochemically with massive proteinuria (> 40 mg/m2/h, or > 50 mg/kg/d), hypoalbuminaemia (< 25 g/l) and hyperlipidemia. Congenital nephrotic syndrome (CNS) usually present in the first 0–3 months of life, which may be caused by congenital syphilis, toxoplasmosis, or congenital viral infections (such as cytomegalovirus). However, majority of CNS cases are caused by monogenic defects of structural proteins that form the glomerular filtration barrier in the kidneys. Mutations of several genes such as NPHS1, NPHS2, WT1, PLCE1, and LAMB2 have been involved [1].. Among the involved genes, mutations in the NPHS1 gene encoding nephrin, are major causes for CNS, which mainly affect exons, but also splice site [2]. According to previously reported cases, none dysmorphic features were present with congenital nephrotic syndrome.

22q11.2duplication syndrome is a chromosomal disease which is a relatively new syndrome with variable clinical features that ranged from normal to mental retardation and with congenital defects [3, 4]. According to published reports, majority of patients with 22q11.2 duplications inherit these from mild- or unaffected parents rather than by de novo mutations, and also none reports have shown that 22q11.2 duplication syndrome with edema, proteinuria, hypoalbuminaemia or hyperlipidemia [3, 5, 6].

Benefiting from rapid development of genetic testing techniques, there have been some rare reports about co-occurrence of two rare genetic disorders in a single patient [7–10], Here, we report a single patient who was caught in CNS as well as 22q11.2 duplication syndrome. Additionally, we performed minigene assay to analyze the effect of the intronic mutation c. 3286 + 5G > A on the splicing of NPHS1 transcript and found this mutation led to aberrant splicing that exon 24 was skipping in the mini gene splicing assay. Based on the results, we verified the pathogenesis of the intronic mutation c.3286 + 5G > A in NPHS1, In all, this report will provide implications for monitoring and highlighting the possibility of co-occurrence of multiple genetic disorders in a single patient.

## Case presentation and methods

### Case presentation

The proband was born at 36 weeks of gestational age spontaneously and weighed 2350 g at birth. He was the second child of the non-consanguineous 20-year-old G2P2 mother and 26-year-old father. There is an elder brother who is 16 months old at the time and develop well according to the father. The mother has moderate learning disability who has self-care ability of daily living but did poorly in the school especially in mathematics. The mother didn’t accept regular prenatal examinations during the pregnancy. Six days after birth, the proband was admitted to our hospital due to fever of 38.5 °C lasting for 6 h.

**Fig. 1:**
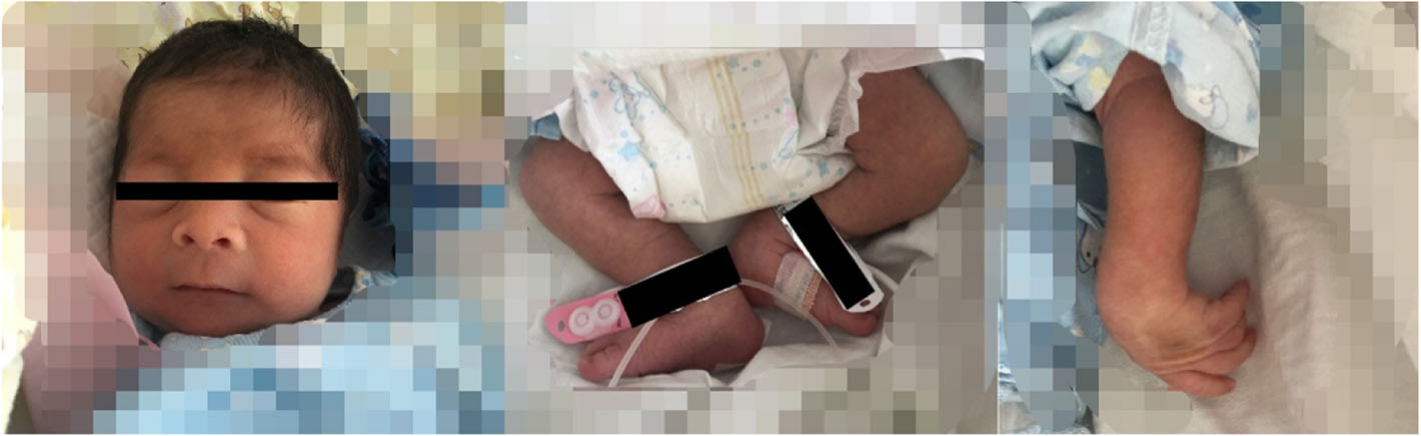
Dysmorphic features of the proband. Hypertelorism, palpebral edema, broad nose bridge, upturned nose, dysmorphic auricle, long philtrum, and a thin upper lip. Additionally, left wrist drop and bilateral strephexopodia, bilateral knee joint flexion contracture

Physical examination at admission time showed dysmorphic features of hypertelorism, palpebral edema, broad nose bridge, upturned nose, dysmorphic auricle, long philtrum, and a thin upper lip. Additionally, we found left wrist drop and bilateral strephexopodia, bilateral knee joint flexion contracture in this patient (Fig. 1).

Laboratory examination revealed hypoproteinemia (total protein 28.4 g/L (65-85 g/L), albumin 13.5 g/L (35-55 g/L), globulin 14.9 g/L (20-40 g/L)), hyperlipemia (triglyceride 2.26 mmol/L (0.3–1.92 mmol/L), cholesterol 7.69 mmol/L (2.32–5.62 mmol/L), low density lipoprotein cholesterin 5.82 mmol/L (1.9–3.12 mmol/L)), heave proteinuria (urine protein 4+ for three times in a week). Further laboratory examination revealed negative for HBsAg, HCV Ab, HIV Ab, TPPA and TORCH Ab. Urinary system ultrasonic testing showed no abnormal. Albumin was used to remit the hypoproteinemia and edema. Finally, the parents refused to accept further therapy and the boy died at age 3 months due to cachexy.

#### Methods

Targeted next generation sequencing (NGS) of the patient was performed with TruSight One Sequencing panel (Illumina, USA) which includes 136 candidate genes associated with hereditary diseases of urinary system. Then a local algorithm (Jinyu medical genetic laboratory, China) was used to explore if there are copy number variations in the area covered by the panel. NGS found two mutations of the NPHS1 gene. Of which, exon18: c.2386G > C; p. (Gly796Arg) was inherited from his mother, intron24: c.3286 + 5G > A; p.? was inherited from his father. NGS also imply a 1.8 Mb dubious duplication on chromosome 22q11.21, which may be the cause of dysmorphic features in the patient. Chromosome microarray analysis (CMA) with the CytoScan750Karray (Affymetrix, USA) was performed to further define the chromosomal aberration and found a 3.2 Mb duplication on chromosome 22q11.21 which is inherited from his mother’s 2.8 Mb duplication at the same region. To clear out whether the intronic mutation c. 3286 + 5G > A is pathogenic, we performed a minigene assay to define the pathogenesis of the intronic variation. We amplified a 1029-bp genomic DNA fragments by PCR, including exon 24, intron 24 and exon 25 of NPHS1 from the proband carrying the c. 3286 + 5G > A variant and a healthy control carrying the wild-type c. 3286 + 5G. The PCR products were ligated to exon trap vector pSPL3 (friendly provided by Prof. Jörg Gromoll). Aberrantly spliced transcripts were identified by sequencing their RT-PCR products with primer SD6 (F) and SA2 (R) (Table 1) that were isolated after electrophoresis through 1.5% agarose gels.

**Table 1:** primers used in minigene assay

Primers	
F	TGTTGCCTAGGCTGGTCTTG
R	GTCCTCTTCCGACCTTCCAG
F′	TTGTGGAGATGGGGGTGGAGATGG
R’	ACACATGGCTTTAGGCTTTGATCCC
SD6	TCTGAGTCACCTGGACAACC
SA2	ATCTCAGTGGTATTTGTCAGC

F and R were used to amplify the genomic fragments of exon 24, intron 24 and exon 25 of *NPHS1* from the proband and healthy control. F′ and R’ were used to amplify the vector pSPL3, SD6 and SA2 were used to amplify and sequence the RT-PCR products from COS7 cells

## Discussion and conclusions

**Fig. 2:**
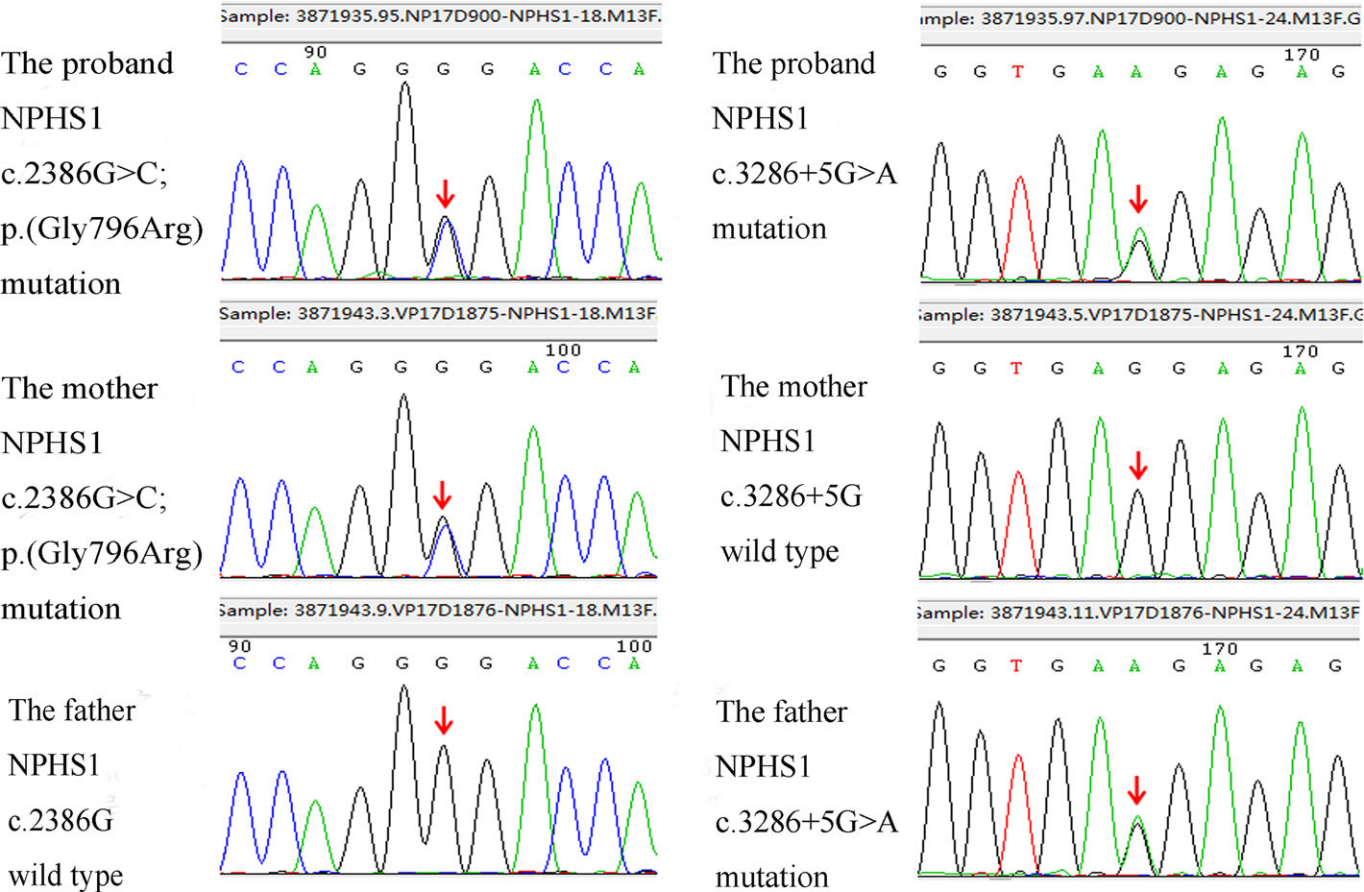
Sanger sequence verification of *NPHS1* mutations found by NGS

**Fig. 3:**
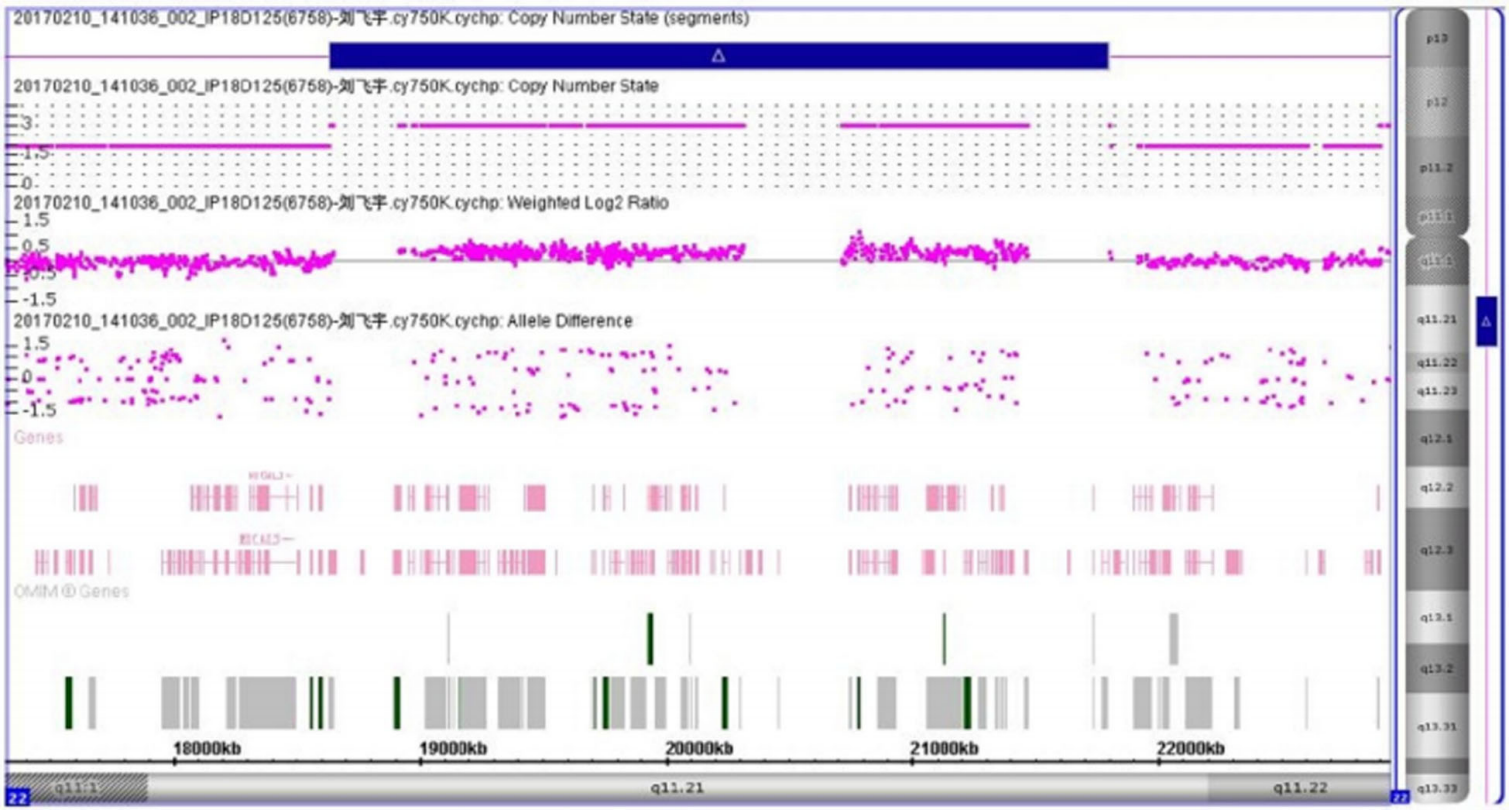
CMA results of the proband with CytoScan 750 K array (Affymetrix, USA), showing a 3.2 duplication on chromosome 22q11.21

In this report, we describe a newborn who was referred to our hospital for fever. At first sight, we noticed palpebral edema and dysmorphic features of face and limbs in this boy (Fig. 1). Laboratory examination supported a diagnosis of CNS. However, dysmorphic features can’t be interpreted with CNS. Therefore, NGS panel with 136 candidate genes associated with hereditary diseases of urinary system as well as CMA were used to detected genetic abnormality of this boy and his parents. NGS revealed mutations of NPHS1 gene which were inherited from his parents respectively (Fig. 2), along with a dubious 1.8 Mb duplication on chromosome 22q11.21. CMA verified that there was a 3.2 Mb duplication on chromosome 22q11.21 which was inherited from his mother’s 2.8 Mb duplication at the same region (Fig. 3).

**Fig. 4:**
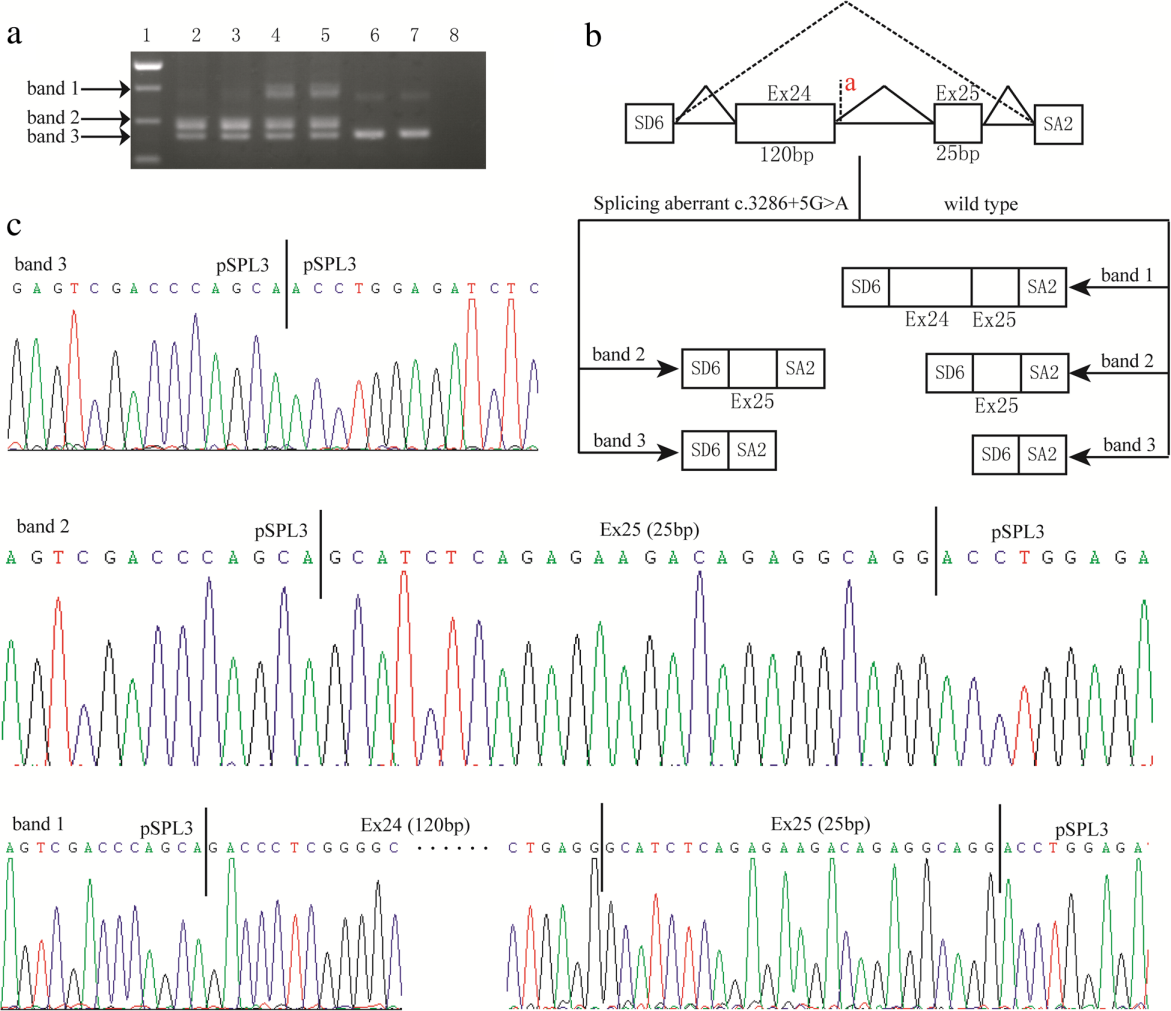
**a** RT-PCR products of the c.3286 + 5G > A in pSPL3 minigene constructs. Lane 1: the 1000 bp marker. Lane 2 and lane 3: the splicing aberrant band. Lane 4 and lane 5: the normal band. Lane 6 and lane 7: empty vector. Lane 8: blank control. **b** Splicing schematic representation of the mini gene vectors used for the in vitro splicing assay. The wild type transcripts have three diverse bands, band 1(407-bp), band 2(288-bp) and band 3(263-bp). Band 1 was the products of exon 24 and exon 25. Band 2 have exon 25. There are no exon in the band 3, which was only the sequence of the pSPL3 vectors. The band 1 was absent in mutation type .The mutation c.3286 + 5G > A led to aberrant splicing that exon 24 was skipping in the mini gene splicing assay. **c** Direct sequencing after gel extraction of the band 1, band 2 and band 3

Mutations of NPHS1 gene cause CNS of the Finnish type, which is a rare autosomal recessive disorder manifesting within the first 3 months of life or even may commencing in utero [11]. Most infants of CNS were premature infants and have a low birthweight for gestational age. The course of CNS is progressive, often leading to end-stage renal disease by 2 or 3 years of age [12]. In this patient, we found two mutations of the NPHS1 gene. Of which, exon18: c.2386G > C; p.(Gly796Arg) was inherited from his mother, and intron24: c.3286 + 5G > A; p.? was inherited from his father. The missense mutation c.2386G > C; p.(Gly796Arg) was ever reported by Buscher et al [13] in diffuse mesangial sclerosis. It was also predicted as a pathogenic mutation with a score of 1.000 by Polyphen, and predicted to affect protein function with a score of 0.05 by SIFT (Table 1). The intronic mutation c.3286 + 5G > A; p.? was ever reported by Wand et al. [14], which is predicted to most probably affect splicing by Human Splicing Finder and MaxEntScan::scoresplice. However, no functional experiments were ever done to verify the pathogenicity of this intronic mutation. In order to further define the pathogenicity of the intronic mutation c.3286 + 5G > A, we performed minigene assay and found that the c.3286 + 5G > A mutation led to aberrant splicing that exon 24 was skipping (Fig. 4). According to edema presented within the first few days of life and typical biochemical results associated with genetic findings and functional analysis of the NPHS1mutations, it’s not hard to draw a diagnosis of CNS.

However, CNS usually have dysmorphic features neither in face nor limbs. So, the dubious duplication on chromosome 22q11.21 implyed by NGS was further verified by CMA with the CytoScan 750 K array (Affymetrix, USA). The parents’ peripheral blood lymphocyte samples were also detected with CMA. We found a 3.2 Mb duplication on chromosome 22q11.21 inherited from his mother’s 2.8 duplication at the same region. Unlike monogenic disorder, 22q11.2 duplication syndrome is a chromosomal disease which is a relatively new syndrome, with variable clinical features that ranged from normal to mental retardation and with congenital defects containing multi cystic dysplastic kidney and esophageal atresia/tracheo-esophageal fistula and vascularring [3, 4, 15, 16]. However, joint contractures found in this patient has never been reported in this syndrome. Because of absence of antenatal data, it is hard to conclude that this is a new manifestation of 22q11.2 duplication syndrome or a consequence of oligohydramnios. The clinical manifestations of 22q11.2 duplication syndrome are of great heterogeneity. However, there are no biochemical abnormalities such as hypoalbuminemia or hypercholesteremia associated with this syndrome according to previously reported cases until now.

This is an interesting case. In a single patient, we found two genetic disorders, CNS and chromosome 22q11.2 duplication syndrome. Although 22q11.2 duplication has been reported in multicystic dysplastic kidney [16], urinary system ultrasonic testing show no abnormal in our patient. In conclusion, we propose that the proband was caught in two different genetic disorder, and this two disorders are independent of each other. This may not be a miracle that a single patient was caught in two or more genetic disorders simultaneously. With the rapid development of genetic detection techniques, new discoveries will increasingly impact our traditional understanding of human genetics.

Due to early death from CNS, we can’t describe more detailed manifestations of 22q11.2 duplication syndrome such as psychomotor and mental development situation in this patient. But obvious dysmorphic features in the proband and moderate learning disability of his mother indicate pathogenicity of this chromosome abnormality in this family. Genetic counseling and prenatal diagnosis are necessary for this family if they plan for another child.

In conclusion, we report a patient who was caught in congenital nephrotic syndrome and 22q11.2 duplication syndrome simultaneously. Report of this patient has implications for monitoring and highlighting the possibility of co-occurrence of multiple genetic disorders in a single patient. Additionally, we found a novel intronic mutation of NPHS1 and functionally defined that the intronic mutation is significant for leading to aberrant splicing, which expand the spectrum of NPHS1 gene mutations.

## Data Availability

The datasets generated and analyzed during the current study are all shown in the manuscript.

## Abbreviations

CMA: Chromosome microarray analysis
CNS: Congenital nephrotic syndrome
NGS: Next generation sequencing
NS: Nephrotic syndrome
